# Serum metabolites may be useful markers to assess vascular invasion and identify normal alpha-fetoprotein in hepatocellular carcinoma undergoing liver resection: a pilot study

**DOI:** 10.1186/s12957-020-01885-w

**Published:** 2020-06-03

**Authors:** Chao-Wei Lee, Ming-Chin Yu, Gigin Lin, Jo-Chu Chiu, Meng-Han Chiang, Chang-Mu Sung, Yi-Chung Hsieh, Tony Kuo, Cheng-Yu Lin, Hsin-I Tsai

**Affiliations:** 1 Division of General Surgery, Department of Surgery, Linkou Chang Gung Memorial Hospital, Taoyuan, Taiwan; 2 Graduate Institute of Clinical Medical Sciences, Chang Gung University, Taoyuan, Taiwan; 3 College of Medicine, Chang Gung University, Taoyuan, Taiwan; 4 Clinical Metabolomics Core Laboratory, Linkou Chang Gung Memorial Hospital, Taoyuan, Taiwan; 5 Department of Medical Imaging and Intervention, Linkou Chang Gung Memorial Hospital, Taoyuan, Taiwan; 6 Imaging Core Laboratory, Institute for Radiological Research, Linkou Chang Gung Memorial Hospital and Chang Gung University, Taoyuan, Taiwan; 7 Department of Gastroenterology and Hepatology, Linkou Chang Gung Memorial Hospital, Taoyuan, Taiwan; 8 Department of Anesthesiology, Linkou Chang Gung Memorial Hospital, Taoyuan, Taiwan

**Keywords:** Metabolomics, Metabolites, Vascular invasion, Formate, Hepatocellular carcinoma, Hepatoma

## Abstract

**Purpose:**

Hepatocellular carcinoma (HCC) is the most common primary malignancy of the liver with a dismal prognosis. Vascular invasion, among others, is the most robust indicator of postoperative recurrence and overall survival after liver resection for HCC. Few studies to date have attempted to search for effective markers to predict vascular invasion before the operation. The current study would examine the plasma metabolic profiling via ^1^H-NMR of HCC patients undergoing liver resection and aim to search for potential biomarkers in the early detection of HCC with normal alpha-fetoprotein (AFP) and the diagnosis of vascular invasion preoperatively.

**Materials and methods:**

HCC patients scheduled to receive liver resections for their HCC were recruited and divided into two separate groups, investigation cohort and validation cohort. Their preoperative blood samples were collected and subjected to a comprehensive metabolomic profiling using ^1^H-nuclear magnetic resonance spectroscopy (NMR).

**Results:**

There were 35 HCC patients in the investigation group and 22 patients in the validation group. Chronic hepatitis B remained the most common etiology of HCC, followed by chronic HCV infection. The two study cohorts were essentially comparable in terms of major clinicopathological variables. After ^1^H-nuclear NMR analysis, we found in the investigation cohort that HCC with normal alpha-fetoprotein (AFP **<** 15 ng/mL) had significantly higher serum level of O-acetylcarnitine than those with higher AFP (AFP ≥ 15 ng/mL, *P* = 0.025). In addition, HCC with microscopic vascular invasion (VI) had significantly higher preoperative serum level of formate than HCC without microscopic VI (*P* = 0.023). These findings were similar in the validation cohort.

**Conclusion:**

A comprehensive metabolomic profiling of HCC demonstrated that serum metabolites may be utilized to assist the early diagnosis of AFP-negative HCC patients and recognition of microvascular invasion in order to facilitate preoperative surgical planning and postoperative follow-up. Further, larger scale prospective studies are warranted to consolidate our findings.

## Introduction

Hepatocellular carcinoma (HCC) is the most common primary malignancy of the liver with an estimated incidence of mortality at 700,000 per year worldwide [1]. In Taiwan, HCC is the second most common cause of cancer death, with more than 8000 deaths annually [2]. Surgical resections remain the most effective therapy in selected patients; however, the extent and feasibility of such are still complicated by the patients’ coexisting liver diseases, such as chronic hepatitis B or C and alcoholic liver disease. Less than 40% of non-cirrhotic HCC patients received liver resections, and the rate was even much lower in patients with chronic liver disease or overt cirrhosis [3]. Regardless a significant improvement in the surveillance program, diagnostic modality, surgical techniques, and postoperative care, the recurrence rate of HCC after liver resection, liver transplantation, or radiofrequency ablation (RFA) still remains as high as 60% [4]. Given the low operative and high recurrence rate of HCC, it is therefore of paramount importance to search for potential markers in the prediction of tumor recurrence or long-term survival after liver resection to ensure optimal surveillance or opportune adjuvant treatment postoperatively.

Risk factors for HCC recurrence after surgery have been identified, including lymph node metastasis (LNM) [5], vascular invasion, poor tumor differentiation, large tumor size, and multinodularity [6, 7], among which, vascular invasion (VI), being the most robust indicator of postoperative recurrence and overall survival [8]. VI can be subdivided into macroscopic VI when larger or major vessels are invaded and detectable by preoperative radiologic study and microscopic VI when small blood vessels are invaded and identified only under microscopic examination. In the presence of preoperative VI, more aggressive surgical treatment such as wider safety margin or anatomical liver resection can be performed. Moreover, when VI is highly suspected, perioperative transarterial chemoembolization (TACE) can be applied. That said, microscopic VI is barely detectable by conventional radiologic studies. Few studies to date have attempted to search for effective markers in the prediction of preoperative microscopic VI. An overexpression of abnormal spindle-like microcephaly associated (ASPM) and upregulation of cytohesin-3 have been found to be associated with VI and early tumor recurrence [9, 10]. Nevertheless, these molecular markers are barely detectable in percutaneous biopsy or before the operation. For real-world clinical practicing, a reliable predictive marker readily available from blood samples may be the best resort.

Metabolomics, a new science that simultaneously detects many metabolites in biofluids and tissues, has been employed in the fields of oncology and various other diseases for the evaluation of their altered levels in a pathological state. Recently, metabolomics has also been employed in the field of HCC in search for diagnostic as well as predictive biomarkers for HCC. HCC patients in different pathological status were found to possess different plasma profiling [11]. For example, Di Poto et al. had demonstrated that a panel consisting of 11 serum metabolites and three clinical factors (AFP, Child–Pugh score, and etiologic factors) had a higher area under the ROC curve compared to AFP alone for the diagnosis of HCC [12, 13]. Luo et al. identified a biomarker panel (phenylalanyl-tryptophan and glycocholate) that had a better diagnostic value compared to AFP for distinguishing HCC patients from a high-risk cohort of cirrhotic patients [14]. Pathways of lysophospholipid, sphingolipid, bile acid, amino acid, and reactive oxygen species metabolism were found to be consistently associated with the development of HCC [15]. Moreover, increased uptake of glucose, enhanced lactate production, and altered lipid metabolism have been defined to be characteristic metabolic signatures in HCC [16]. As for the prediction of outcome, by using metabolomic platforms, ethanolamine, lactic acid, acotinic acid, phenylalanine, and ribose were found useful in discriminating patients with early recurrence from those without recurrence [17], and metabolomic fingerprints rather than tumor size appeared to show an impact on poor prognosis [18]. Lately, Zhou et al. [19] identified that a combination of serum methionine, GCDCA, and cholesterol sulfate levels could predict early HCC recurrence with high accuracy. Despite these remarkable results, few studies to date have tried to search for potential metabolites predictive of microscopic VI in HCC. In this study, we present plasma metabolic profiling via ^1^H-nuclear magnetic resonance spectroscopy (^1^H-NMR) of HCC patients undergoing liver resection and aim to search for potential biomarkers in the early detection of HCC with normal alpha-fetoprotein (AFP) and the diagnosis of VI preoperatively.

## Materials and methods

### Patient selection

Under the approval of the Institutional Review Boards of Chang Gung Memorial Hospital (CGMH), primary HCC patients scheduled to receive curative liver resection for their tumors were included into our study. After informed consent was completed, 10 ml of blood was drawn both before the operation and 1 month after the operation. The patients were asked to stay fasted prior to blood tests. The serum was extracted by centrifugation and was stored into aliquots and frozen at − 80 °C until further batch analysis by hydrogen-1 nuclear magnetic resonance (^1^H-NMR) in the Metabolomic Core Laboratory at CGMH and Chang Gung University (CGU). Patient demographic data including age, gender, cigarette smoking, alcohol consumption, hepatitis B virus (HBV) infection, anti-hepatitis C virus antibody (anti-HCV) level, serum bilirubin, albumin, prothrombin levels, international normalized ratio (INR), serum alanine aminotransferase (ALT) activity, serum aspartate aminotransferase (AST) activity, serum gamma-glutamyltransferase (GGT) activity, platelet count, and alpha-fetal protein (AFP) level were collected. Their pathological factors and oncological outcome including tumor lymph node metastasis status, tumor encapsulation, histological grade, VI, daughter nodules, resection margin, fatty change, liver cirrhosis, tumor recurrence, and long-term survival were also recorded. Early recurrence is defined as the occurrence of tumor recurrence within 2 years of the curative operation. Overall survival (OS) is defined by the time elapsing from the date of diagnosis to either the date of death or the date of the last contact. Cases with surgical mortality, defined as death within 1 month of surgery were excluded from the survival analyses. A separate cohort was also recruited for validation.

### NMR spectroscopy analysis

#### General procedure

The procedures briefly described below were aimed to obtain high-quality information for metabolic study, including (a) chemical shifts (value, TMS, or TSP as 0.0), (b) spin-spin coupling and coupling constants (J value), (c) relaxation (may not necessary all times), and (d) signal intensity (for quantitation). The standardized procedure currently used in the Metabolomic Core Laboratory, CGU, is a modified protocol of that described by Beckonert et al. [20].

#### NMR measurement

##### Measurement of the ^1^H-NMR spectra for aqueous extracts

A NOESY pulse sequence with water irradiation during the relaxation delay and during the mixing time were used (RD-90^°^-t-90^°^-tm-90^°^-ACQ) (RD is the relaxation delay, t is a short delay typically of about 3 s, 90^°^ represents a 90^°^ RF pulse, tm is the mixing time, and ACQ was the data acquisition period. The mixing time is tm = 100–150 ms). Other parameters are as follows: spectral width = 20 ppm, number of time domain data points = 32,768, relaxation delay, RD = 2.0 s, acquisition time = 1.36 s, number of scans = 64 for urine and 128 for plasma, serum, and tissue extracts. This resulted in a total acquisition time of about 8–12 min per sample (128 scans) [20]. The pulse sequence was suitable for NMR signal quantitation.

##### Lipophilic metabolites (lipidomics)

The ^1^H-NMR spectra of lipid extracts did not require water suppression. A routine 90^°^ pulse sequence was sufficient in measuring fully relaxed spectra (for quantitation purpose). In this core laboratory, a routine collect 64 transients (NS) into 32,768 data points was carried out. A relaxation delay = 2 s, spectral width = 20 ppm, and acquisition time = 1.36 s can be used [20]. For quantitation, signal intensity was used. A mixture of lipid metabolites containing known amounts of free cholesterol and cholesterol oleate, phospholipids, and TG were used as reference sample. The crude lipid extracts (Folch method) of a mixture of plasma samples were used for calibration.

##### NMR data management

The conversion of FID signals to spectral data was carried out using the softwares of the available software package of NMR instruments. Assignment of the chemical shifts to specific metabolites will base on spectral data in the literature or by the information based on the measurement of authentic metabolites in our chemical library. An NMR data base (not complete) of LMW metabolites (pH adjusted) was available. Several non-commercial and commercial application softwares such as *MetaboAnalyst* [21] were used for metabolite identification. The NMR spectrum was normalized by internal standard (TSP, 3-(Trimethylsilyl)propionic-2,2,3,3-d4 acid sodium salt). The missing value of the identified metabolites was defined as zero.

### Statistical analysis

The statistical analysis was performed with IBM SPSS Statistics 21 (IBM Corporation, Software Group, Somers, NY, USA). Fisher’s exact test and Pearson’s χ2 test were used to analyze categorical data. Student’s *t* test was used to analyze quantitative variables. Statistical significance is defined as *P* < 0.05.

## Results

### Patient characteristics

A total of 35 patients were enrolled to the study as the investigation group. Their blood samples were collected the day before and 1 month after the operation. As shown in Table 1, of the 35 patients in the investigation cohort, the majority were males, with an average of 62.7 ± 1.9 years of age. Twenty-three patients (65.7%) had HBV infection and 8 (22.9%) had HCV infection. The majority of the investigation group had serum AFP levels greater than 15 ng/mL, with an average of 16,096 ± 12,568 ng/mL (Table 2). Nine patients received major surgical procedures, and about 70% of tumors were less than 5 cm in diameter. Among the investigation group, 20% was non-encapsulated HCC, 40% had VI, 11.4% had daughter nodules, 8.6% was ruptured tumor, and 40% had pathologically proven cirrhosis. Using Edmondson and Steiner grade, the distribution of grade I to IV HCC in the investigation cohort was 11.8%, 28.6%, 52.9%, and 8.8%, respectively. Another 22 patients were recruited as the validation cohort with similar clinicopathological characteristics as the investigation cohort, except all 22 patients in the validation cohort were encapsulated HCC (Table 2).

**Table 1:** Patient demographics and tumor characteristics of the investigation cohort (*n* = 35)

	No (%)
**Age (year) (mean 62.7 ± 1.9)**	
≦ **65**	20 (57.1)
> **65**	15 (42.9)
**Gender**	
**Male**	27 (77.1)
**Female**	8 (22.9)
**Smoking**	
**Yes**	11 (31.4)
**No**	24 (68.6)
**Alcohol**	
**Yes**	6 (17.1)
**No**	29 (82.9)
**HBsAg**	
**Positive**	23 (65.7)
**Negative**	12 (34.3)
**Anti-HCV Ab**	
**Positive**	8 (22.9)
**Negative**	27 (77.1)
**Cirrhosis**	
**Yes**	14 (40.0)
**No**	21 (60.0)
**ICG-15 (%)**	
**≦ 10**	20 (57.1)
**> 10**	15 (42.9)
**AFP (ng/ml)**	
**≦ 15**	14 (40.0)
**> 15**	21 (60.0)
**AFP (ng/ml)**	
**≦ 200**	27 (77.1)
**> 200**	8 (22.9)
**Surgical procedure**	
**Major**	9 (25.7)
**Minor**	26 (74.3)
**Size (cm)**	
**≦ 5**	24 (68.6)
**> 5**	11 (31.4)
**Encapsulation**	
**Yes**	28 (80.0)
**No**	7 (20.0)
**Vascular invasion**	
**Yes**	14 (40.0)
**No**	21 (60.0)
**Tumor rupture**	
**Yes**	3 (8.6)
**No**	32 (91.4)
**Daughter nodules**	
**Yes**	4 (11.4)
**No**	31 (88.6)
**Resection margin**	
**Positive**	0 (0)
**Negative**	35 (100)
**Edmondson grade**	
**I/II**	4(11.8)/10(28.6)
**III/IV**	18(52.9)/3(8.8)
**Early recurrence**^a^	
**Yes**	4 (11.4)
**No**	31 (88.6)

^a^Tumor recurrence within 2 years after surgery

**Table 2:** Comparison of clinicopathological characteristics between investigation cohort (*n* = 35) and validation cohort (*n* = 22)

	Categorical variables (***n*** (%))				Continuous variables (mean ± SE)		
	Investigation	Validation	***P*** value		Investigation	Validation	***P*** value
**Male gender**	27 (77.1)	19 (86.4)	0.502	**Age (year)**	62.7 ± 1.9	60.2 ± 2.4	0.429
**HBV infection**	23 (65.7)	11 (50.0)	0.277	**BMI (Kg/m**^**2**^**)**	23.9 ± 0.5	25.6 ± 0.7	0.063
**HCV infection**	8 (22.9)	9 (40.9)	0.234	**ICG-15 (%)**	9.53 ± 0.88	11.44 ± 1.64	0.316
**AFP > 15 ng/mL**	21 (60.0)	10 (45.5)	0.413	**AFP (ng/mL)**	16096 ± 12568	17938 ± 17777	0.931
**Major procedure**	9 (25.7)	6 (27.3)	1.000	**CEA (ng/mL)**	6.07 ± 4.07	1.57 ± 0.20	0.361
**Encapsulation**	28 (80.0)	22 (100.0)	0.036	**CA 19-9 (U/mL)**	26.6 ± 3.7	22.1 ± 3.9	0.421
**Vascular invasion**	14 (40.0)	10 (45.5)	0.785	**Size (cm)**	4.59 ± 0.61	4.05 ± 0.68	0.557
**Daughter nodules**	4 (11.4)	2 (9.1)	1.000				
**Tumor rupture**	3 (8.6)	1 (4.5)	1.000				
**Cirrhosis**	14 (40.0)	13 (59.1)	0.184				
**Edmondson and Steiner Grade (I/II/III/IV)**	4(11.8)/10(28.6)/18(52.9)/3(8.8)	3(13.6)/13(59.1)/5(22.7)/1(4.5)	0.073				

### NMR plasma profiles in HCC patients in relation to serum AFP levels

**Fig. 1:**
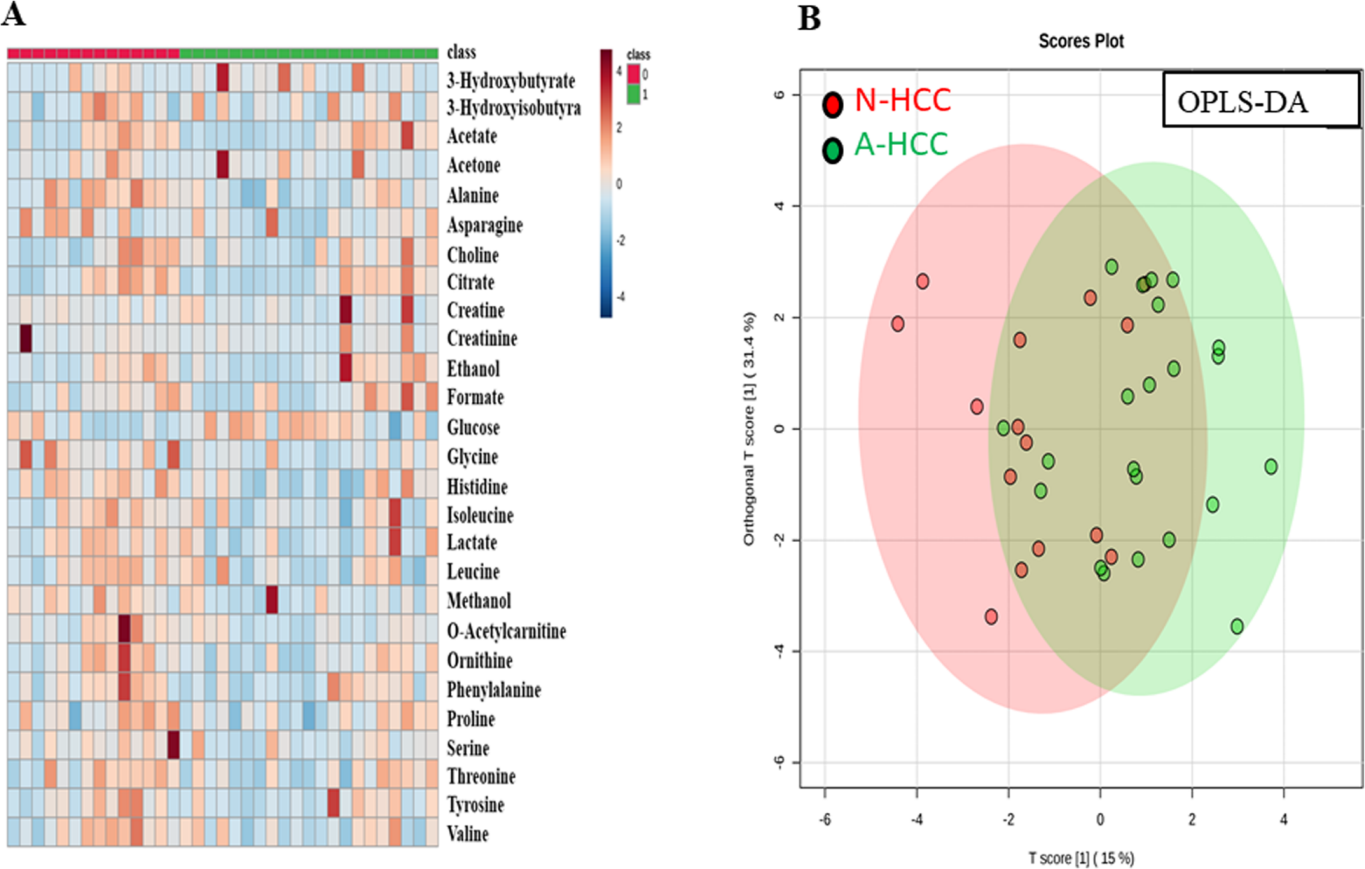
Metabolome analysis by NMR in HCC. **a** Heatmap analysis in HCC with or without normal AFP. **b**^1^H-NMR plasma profiles using OPLS-DA. A separation is seen between the two groups. Red area denotes HCC patients with normal AFP levels (less than 15 ng/mL, N-HCC) while green area denotes HCC patients with abnormal AFP levels (greater or equal to 15 ng/mL, A-HCC)

As demonstrated in Fig. 1, heatmap showed the clustering of metabolites that highly correlated with the status of HCC in the absence of AFP elevation. Also, ^1^H-NMR plasma profiles using OPLS-DA, in which red area denoted HCC patients with normal AFP levels (less than 15 ng/mL) while green area denoted HCC patients with abnormal AFP levels (greater or equal to 15 ng/mL), have shown a separation between the patients with normal (< 15 ng/mL) and elevated AFP (≥ 15 ng/mL).

Further analysis on the identifications of metabolites associated with AFP levels revealed a few metabolites possibly responsible for the group separation, including glycine, O-acetylcarnitine, histidine, and ornithine. Of particular interest, the level of O-acetylcarnitine was much higher in the normal AFP-HCC group (N-HCC) than abnormal AFP-HCC group (A-HCC) with statistical significance (0.600 ± 0.08 vs*.* 0.374 ± 0.11, *P* value 0.025). Similar trend was observed in the validation group in which the level of O-acetylcarnitine was 0.958 ± 0.09 vs. 0.687 ± 0.08 in N-HCC and A-HCC, respectively, as shown in Table 3.

**Table 3:** Nuclear magnetic resonance (NMR) observed serum metabolites with intensity differences associated with different AFP for hepatocellular carcinoma

Metabolites	AFP < 15 ng/ml	AFP ≥ 15 ng/ml	***P*** value^a^
**Investigation cohort (*n*= 35)**			
Glycine	26.128 ± 2.83	18.627 ± 1.09	0.006
O-Acetylcarnitine	0.600 ± 0.08	0.374 ± 0.11	0.025
Histidine	6.597 ± 0.36	5.532 ± 0.37	0.062
Ornithine	9.263 ± 1.05	7.065 ± 0.58	0.073
**Validation cohort (*n*= 22)**			
Glycine	26.810 ± 1.33	27.195 ± 2.19	0.752
O-Acetylcarnitine	0.958 ± 0.09	0.687 ± 0.08	0.032
Histidine	7.303 ± 0.32	6.070 ± 0.55	0.153
Ornithine	10.504 ± 0.86	9.061 ± 0.75	0.215

Metabolites were expressed as mean ± SE in arbitrary units

^a^Student’s *t* test

**Fig. 2:**
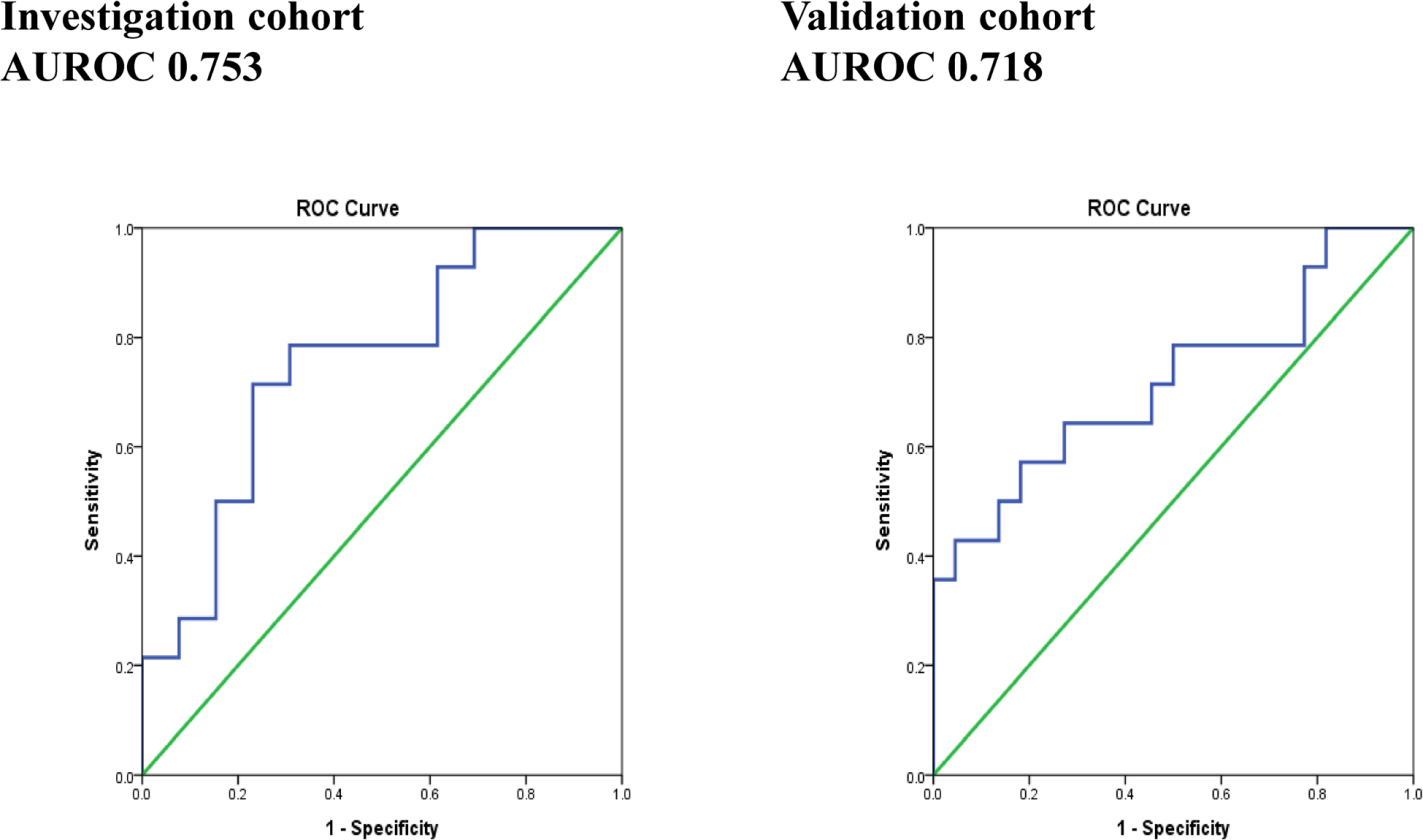
ROC curves of O-acetylcarnitine for HCC with normal AFP. In the entire HCC group, O-acetylcarnitine has shown an area under the curve (AUC) of the receiver-operating characteristics (ROC) or AUROC of 0.753 in the investigation group and an AUROC of 0.718 in the validation group for N-HCC

The diagnostic ability of O-acetylcarnitine in discriminating N-HCC from the entire HCC cohort was demonstrated by the ROC curves in Fig. 2. In terms of N-HCC, O-acetylcarnitine has shown an area under the curve (AUC) of the receiver-operating characteristics (ROC) or AUROC of 0.753 in the investigation group and an AUROC of 0.718 in the validation group.

### NMR plasma profiles in HCC patients in relation to VI

**Fig. 3:**
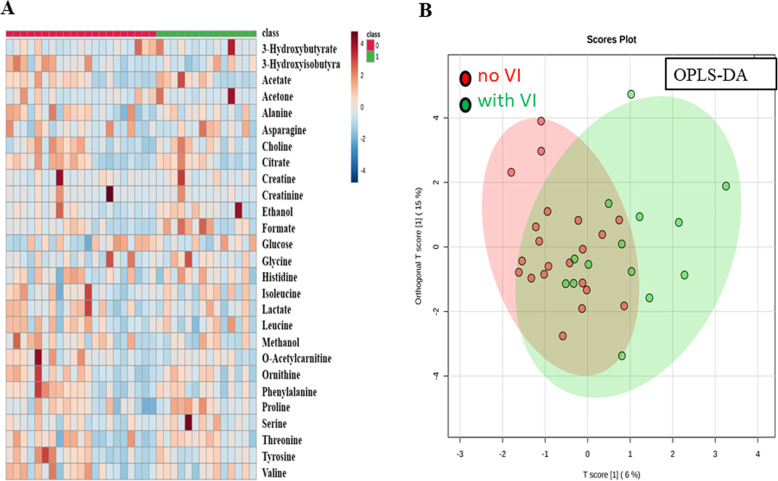
Metabolome analysis by NMR in HCC in association with vascular invasion. **a** Heatmap analysis with or without microscopic vascular invasion. **b**^1^H-NMR plasma profiles using OPLS-DA. A separation is seen between the two groups. Red area denotes HCC patients without vascular invasion while green area denotes HCC patients with vascular invasion

**Fig. 4:**
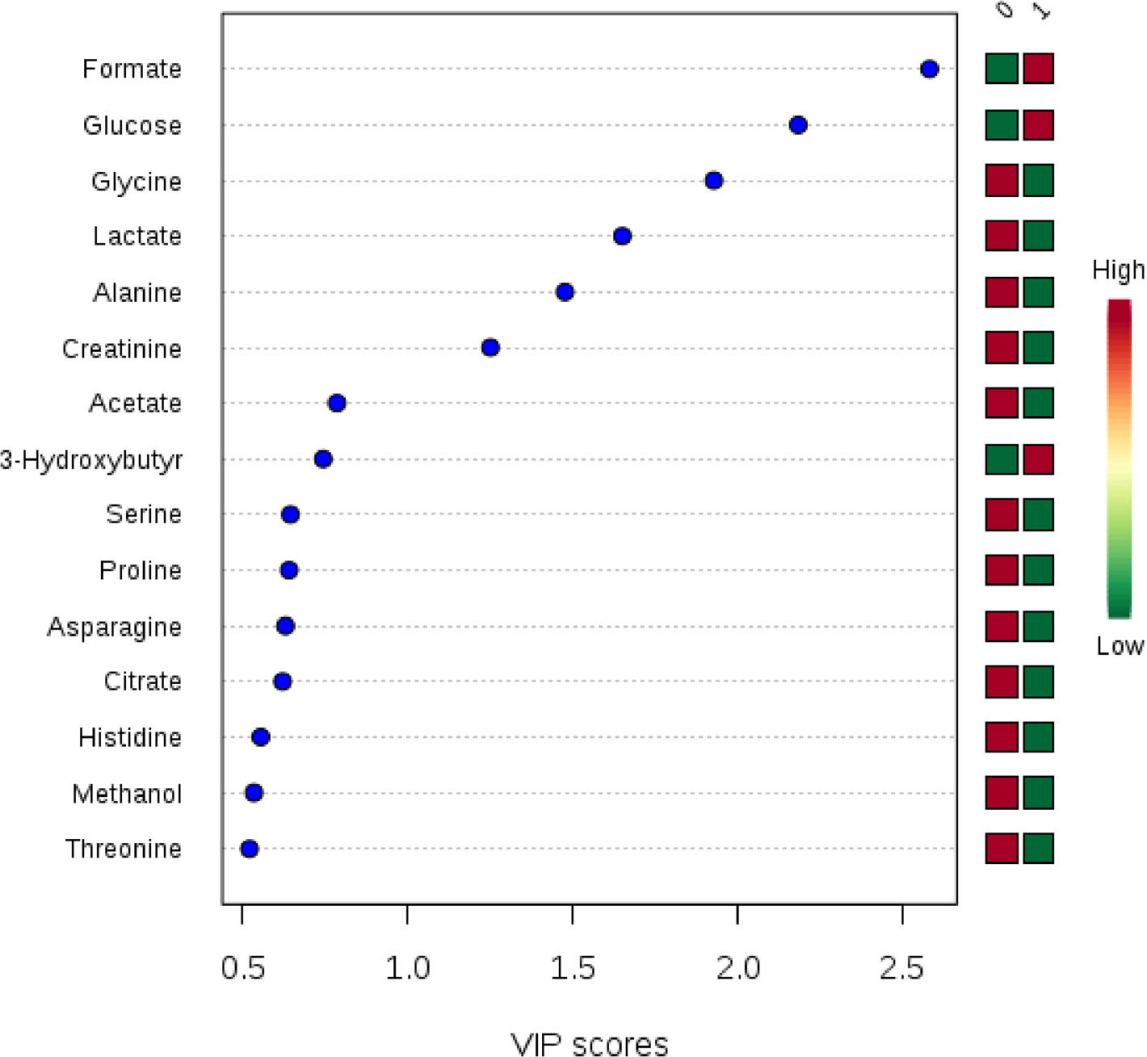
PLS-DA variable importance projection (VIP) scores. The metabolites responsible for the classification of VI or not would be identified using the variable importance in projection (VIP) scores. Metabolites with high VIP are more important in providing class separation, while those with small VIP provide less contribution [22]. Formate had the highest VIP score in terms of VI. Red or green on the right of Fig. 4 indicated the low or high concentration of metabolites by comparing the concentration of each metabolite in patients with (1) or without (0) vascular invasion

As VI is one important prognostic factor in HCC and can potentially alter the extent of surgical treatment, ^1^H-NMR was conducted in an attempt to distinguish the status of microvascular invasion in HCC patients preoperatively. In Fig. 3, heatmap and OPLS-DA demonstrated a separation of metabolites contributing to the microvascular invasion. Red and green denoted the absence and the presence of microvascular invasion. From this model, few metabolites with high variable importance projection (VIP) scores were selected. In Fig. 4, formate appeared to contribute to the separation with the highest VIP score [22]. The level of formate was significantly higher in HCC with VI than HCC without VI (37.592 ± 7.83 vs*.* 19.697 ± 3.23, *P* value 0.023) (Table 4). Similar trend was observed in the validation group in which the level of formate was 18.776 ± 0.99 vs. 14.807 ± 1.48 in HCC with and without VI, respectively.

**Table 4:** Clinical variables and nuclear magnetic resonance (NMR) observed serum metabolites with intensity differences associated with vascular invasion (VI) for hepatocellular carcinoma

Metabolites	Without VI	With VI	***P*** value^a^
**Investigation cohort (*n*= 35)**			
Tumor size (cm)	3.47 ± 0.49	6.29 ± 1.22	0.047
AFP (ng/ml)	6541.91 ± 6486.76	30428.30 ± 30156.35	0.360
ICG-15 (%)	9.80 ± 1.10	9.11 ± 1.54	0.711
Formate	19.697 ± 3.23	37.592 ± 7.83	0.023
**Validation cohort (*n*= 22)**			
Tumor size (cm)	3.80 ± 0.98	4.33 ± 0.95	0.710
AFP (ng/ml)	145.82 ± 87.38	39289.31 ± 39109.13	0.343
ICG-15 (%)	13.18 ± 2.42	9.35 ± 2.10	0.256
Formate^b^	14.807 ± 1.48	18.776 ± 0.99	0.042

Data were expressed as mean ± SE

^a^Student’s *t* test

^b^Expressed in arbitrary units

### Discriminative ability of clinical parameters and biomarker for VI

**Fig. 5:**
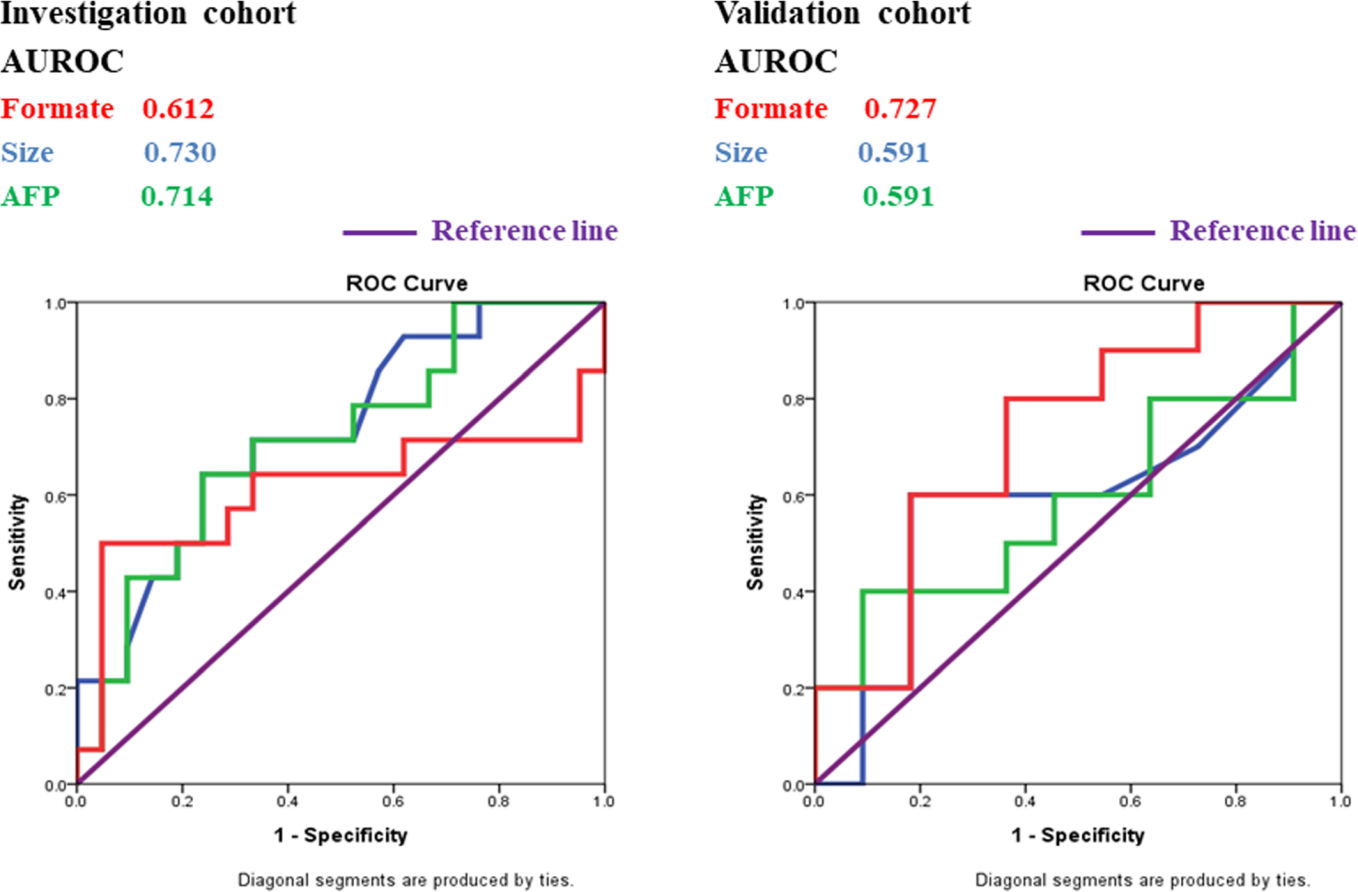
ROC curves of formate, tumor size, and AFP in diagnosing vascular invasion in HCC. Formate has shown an AUROC of 0.612 in the investigation group and an AUROC of 0.727 in the validation group in terms of predicting VI. Tumor size and AFP levels, on the other hand, have AUROC of 0.730 and 0.714 in the investigation group, and 0.591 and 0.591 in the validation group, respectively, for predicting VI

**Fig. 6:**
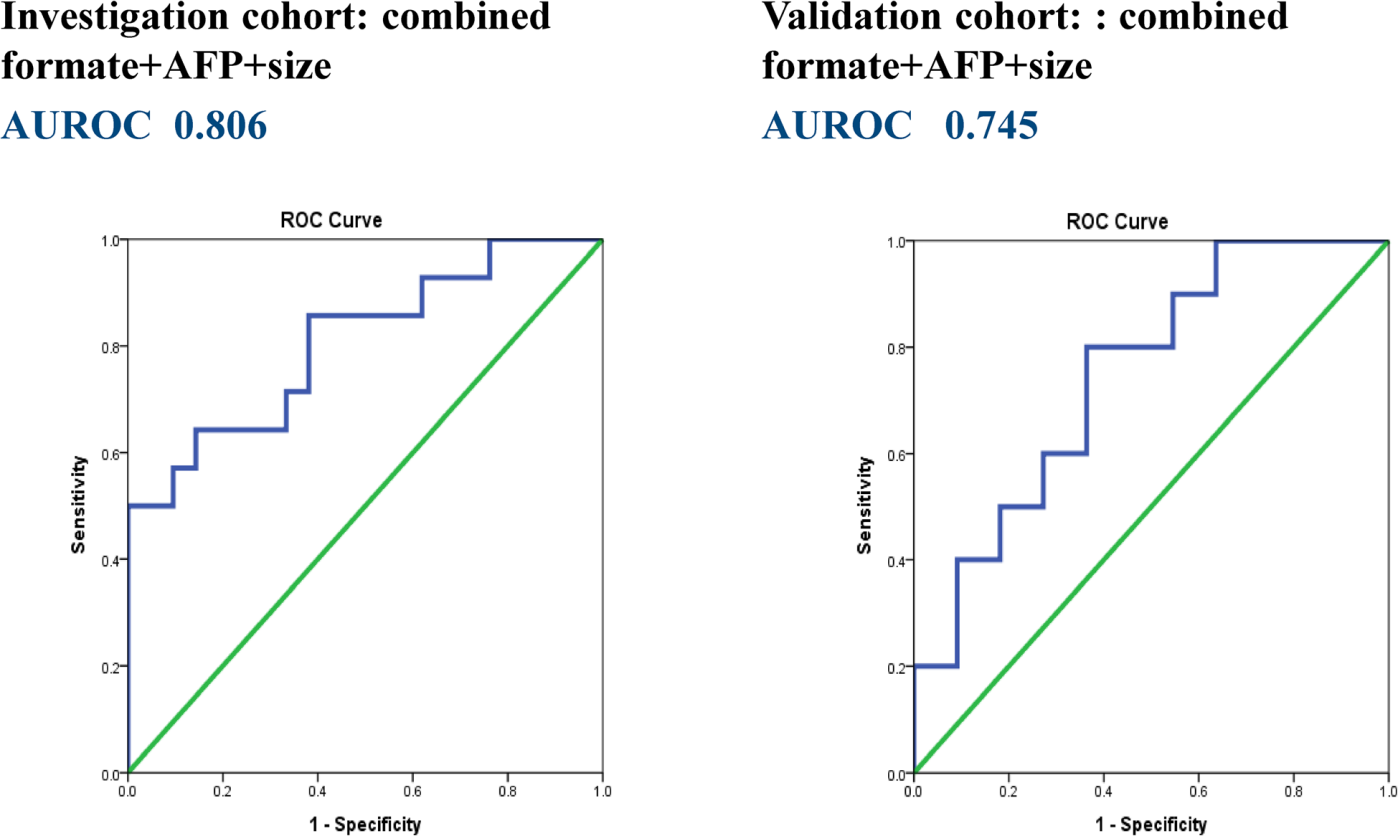
Prediction of vascular invasion in HCC using a combination of formate, tumor size, and AFP. When combining formate, AFP, and tumor size, the AUROC in predicting microscopic VI raised to 0.806 in the investigation group and 0.745 in the validation group

As previously demonstrated, formate was highly selective for the presence of microvascular invasion in HCC patients. Tumor size, serum AFP, and ICG-15 were compared between HCC patients with and without microscopic VI. As shown in Table 4 and Fig. 5, even though tumor size, AFP, and ICG-15 individually may not be adequate in distinguishing the presence of VI, formate appeared to display an acceptable AUROC of 0.612 in the investigation group and 0.727 in the validation group. In combination of formate, AFP, and tumor size, the AUROC in predicting microscopic VI raised to 0.806 in the investigation group and 0.745 in the validation group (Fig. 6).

## Discussions

HCC is one of the most common malignant tumors, with increasing incidence and high mortality rate, in the world [23, 24]. Clinically, HCC is diagnosed by tissue-based histopathological findings, characteristic radiologic features, or blood-based assays such as AFP, and a majority of HCC are diagnosed at an advanced stage. It appears that the sensitivity and specificity of such clinically used methodology may not be adequate in identifying early stage HCC and thus significantly reducing the reliability. Recently, metabolomics has gained its popularity in the field of surgical oncology as homeostasis perturbations related to cancer development may be detected with the new platform. As an extreme complex organ responsible for glycolysis and metabolisms of carbohydrates, amino acids, and fatty acids, literature has revealed that metabolomics based on NMR could be used to identify biomarkers in varying stages of liver diseases [25] in hope for early tumor detection.

AFP level is the most frequently used tumor marker for HCC; however, in the absence of AFP elevation, the diagnosis can be difficult. Metabolomic analysis comparing HCC and healthy individuals has demonstrated that an increased level of acetylcarnitine is associated with HCC and highly correlated with tumor grade [26–28]. In consistency with the literature, we also found elevated serum acetylcarnitine, more so in these normal AFP HCC patients than those with abnormal AFP. Among HCC patients, acetylcarnitine showed a discriminative accuracy of > 70% for those with normal AFP (AFP < 15 mg/L), suggesting that acetylcarnitine may be served as a sensitive and specific biomarker, in complementary to AFP, for monitoring the development of HCC. Acetylcarnitine is an acetylated form of l-carnitine mainly used for energy production by transporting activated long chain fatty acids from the cytosol into mitochondria. Carnitine is naturally produced by the body. As advanced stage of HCC often predisposes patients to cancer cachexia, low serum levels of acetylcarnitine are mainly the result of reduced synthesis of carnitine in HCC patients. Further studies are warranted to elucidate the roles of acetylcarnitine in the diagnosis and follow-up of HCC patients.

In clinical practice, the presence of VI may influence the surgeons’ decision in terms of the extent of resection. VI is found to be associated with the presence of ascites in patients with HBV-related cirrhotic HCC [29]. Other evidence has also shown that derived neutrophil to lymphocyte ratio (dNLR), lymphocyte-to-monocyte ratio (LMR), age, and tumor size were independent predictors of micro-vascular invasion [30]. Kurokawa T et al. [31] discovered that next generation des-r-carboxy prothrombin (NX-DCP) may be used to predict the presence of VI in HCC. However, these researches are mostly correlation studies, and they failed to explain the mechanism underlying the association between the markers and VI. Moreover, most of them did not have validation cohorts to confirm their findings in the investigation cohort. As a result, it is of urgent need to develop a reliable biomarker to predict and explain VI prior to liver resection.

Several recent studies have demonstrated that formate is a useful biomarker in HCC [32–34]; however, what we have shown is that formate itself is a highly specific and selective biomarker for the prediction of VI and a combination of formate, tumor size and AFP further improves its performance. Our finding is supported by the study done by Meiser et al. that an increased formate overflow is a hallmark of oxidative cancers and that high formate levels promote tumor invasion [35]. Furthermore, they also discovered that inhibition of formate production by genetic interference could reduce cancer cell invasion. Their study is remarkable and further confirmed our finding that formate may be an effective serum marker to predict VI in HCC. Moreover, from the study mentioned above, the influence of formate is potentially targetable and may be utilized to develop treatment strategies against HCC with VI.

The results of the current study have shown that these metabolic changes are a result of carcinogenesis related to energy metabolism, lipid metabolism, and tricarboxylic acid (TCA) cycle. Given the small sample size and multiple comparisons, interpretation of the results must be cautious, even with validation data. As liver is a complex organ also responsible for lipid and amino acid metabolism, the use of high throughput liquid chromatography and mass spectrometry such as liquid chromatography mass spectrometry (LC-MS) with larger sample size is required.

## Conclusion

In conclusion, metabolomic profiling can identify novel biomarkers that assist in the early diagnosis of AFP-negative HCC patients and recognition of microvascular invasion in order to facilitate preoperative surgical planning and postoperative follow-up. Further, larger scale prospective cohort studies are warranted to consolidate our findings.

## Data Availability

All data generated or analyzed during the study are included in this published article. Raw data may be requested from the authors with the permission of the institution.

## Abbreviations

ACQ: Acquisition period
A-HCC: HCC with elevated or abnormal AFP levels (greater or equal to 15 ng/mL)
AFP: Alpha-fetoprotein
ALT: Alanine aminotransferase
AJCC: American Joint Committee on Cancer
ASPM: Abnormal spindle-like microcephaly associated
AST: Aspartate aminotransferase
AUC: Area under the curve
AUROC: Area under the curve of the receiver-operating characteristics
BMI: Body mass index
CA 19-9: Cancer antigen 19-9
CEA: Carcinoembryonic antigen
CGMH: Chang Gung Memorial Hospital
CGU: Chang Gung University
CI: Confidence intervals
CT: Computed tomography
DM: Diabetes mellitus
ECOG: Eastern Cooperative Oncology Group
GGT: Gamma-glutamyltransferase
GCDCA: Glycochenodeoxycholic acid
HBV: Hepatitis B virus
HCC: Hepatocellular carcinoma
HCV: Hepatitis C virus
^1^H-NMR: Hydrogen-1 nuclear magnetic resonance
HR: Hazard ratios
ICG-15: Indocyanine green retention test at 15 min
INR: International normalized ratio
LC-MS: Liquid chromatography mass spectrometry
LNM: Lymph node metastasis
MRI: Magnetic resonance imaging
N-HCC: HCC with normal AFP levels (less than 15 ng/mL)
NX-DCP: Next generation des-r-carboxy prothrombin
OPLS-DA: Orthogonal partial least squares-discriminant analysis
OS: Overall survival
RD: Relaxation delay
RFA: Radiofrequency ablation
ROC: Receiver-operating characteristics
TACE: Transarterial chemoembolization
TCA: Tricarboxylic acid
tm: Mixing time
TMS: Tetramethylsilane
TSP: 3-(trimethylsilyl)-propionate, sodium salt
VI: Vascular invasion
VIP: Variable importance projection

## References

[1] Torre LA, Bray F, Siegel RL, Ferlay J, Lortet-Tieulent J, Jemal A (2015). Global cancer statistics, 2012. CA: a cancer journal for clinicians..

[2] Department of Health ROC. Report of leading cancer-related death in 2014. *Department of Health, Executive Yuan, Rebpulic of China.* 2015.

[3] Forner A, Llovet JM, Bruix J. Hepatocellular carcinoma. *Lancet (London, England).* Mar 31 2012;379(9822):1245-1255. 10.1016/S0140-6736(11)61347-0 PMC13537732 22353262

[4] Tung-Ping Poon R, Fan ST, Wong J (2000). Risk factors, prevention, and management of postoperative recurrence after resection of hepatocellular carcinoma. Ann Surg..

[5] Lee CW, Chan KM, Lee CF (2011). Hepatic resection for hepatocellular carcinoma with lymph node metastasis: clinicopathological analysis and survival outcome. Asian J..

[6] Shah SA, Cleary SP, Wei AC (2007). Recurrence after liver resection for hepatocellular carcinoma: risk factors, treatment, and outcomes. Surgery..

[7] Ibrahim S, Roychowdhury A, Hean TK (2007). Risk factors for intrahepatic recurrence after hepatectomy for hepatocellular carcinoma. Am J Surg..

[8] Tandon P, Garcia-Tsao G (2009). Prognostic indicators in hepatocellular carcinoma: a systematic review of 72 studies. Liver international : official journal of the International Association for the Study of the Liver..

[9] Lin SY, Pan HW, Liu SH, et al. ASPM is a novel marker for vascular invasion, early recurrence, and poor prognosis of hepatocellular carcinoma. *Clinical cancer research : an official journal of the American Association for Cancer Research.* Aug 1 2008;14(15):4814-4820. 10.1158/1078-0432.CCR-07-5262 18676753

[10] Fu Y, Li J, Feng MX (2014). Cytohesin-3 is upregulated in hepatocellular carcinoma and contributes to tumor growth and vascular invasion. International journal of clinical and experimental pathology..

[11] Chen Y, Zhou J, Li J, Feng J, Chen Z, Wang X. Plasma metabolomic analysis of human hepatocellular carcinoma: diagnostic and therapeutic study. *Oncotarget.* Jun 17 2016. 10.18632/oncotarget.10119 PMC5216945 27322079

[12] Liu XN, Cui DN, Li YF, Liu YH, Liu G, Liu L. Multiple “Omics” data-based biomarker screening for hepatocellular carcinoma diagnosis. *World J Gastroenterol.* Aug 14 2019;25(30):4199-4212. 10.3748/wjg.v25.i30.4199 PMC6700689 31435173

[13] Di Poto C, Ferrarini A, Zhao Y (2017). Metabolomic characterization of hepatocellular carcinoma in patients with liver cirrhosis for biomarker discovery. Cancer epidemiology, biomarkers & prevention : a publication of the American Association for Cancer Research, cosponsored by the American Society of Preventive Oncology..

[14] Luo P, Yin P, Hua R (2018). A large-scale, multicenter serum metabolite biomarker identification study for the early detection of hepatocellular carcinoma. Hepatology..

[15] Fitian AI, Cabrera R. Disease monitoring of hepatocellular carcinoma through metabolomics. *World journal of hepatology.* Jan 8 2017;9(1):1-17. 10.4254/wjh.v9.i1.1 PMC5220267 28105254

[16] De Matteis S, Ragusa A, Marisi G (2018). Aberrant metabolism in hepatocellular carcinoma provides diagnostic and therapeutic opportunities. Oxidative medicine and cellular longevity..

[17] Ye G, Zhu B, Yao Z, et al. Analysis of urinary metabolic signatures of early hepatocellular carcinoma recurrence after surgical removal using gas chromatography-mass spectrometry. *Journal of proteome research.* Aug 3 2012;11(8):4361-4372. 10.1021/pr300502v 22768978

[18] Nahon P, Amathieu R, Triba MN, et al. Identification of serum proton NMR metabolomic fingerprints associated with hepatocellular carcinoma in patients with alcoholic cirrhosis. *Clinical cancer research : an official journal of the American Association for Cancer Research.* Dec 15 2012;18(24):6714-6722. 10.1158/1078-0432.CCR-12-1099 23136190

[19] Zhou L, Liao Y, Yin P, et al. Metabolic profiling study of early and late recurrence of hepatocellular carcinoma based on liquid chromatography-mass spectrometry. *Journal of chromatography. B, Analytical technologies in the biomedical and life sciences.* Sep 1 2014;966:163-170. 10.1016/j.jchromb.2014.01.057 24582150

[20] Beckonert O, Keun HC, Ebbels TM (2007). Metabolic profiling, metabolomic and metabonomic procedures for NMR spectroscopy of urine, plasma, serum and tissue extracts. Nature protocols..

[21] Xia J, Psychogios N, Young N, Wishart DS. MetaboAnalyst: a web server for metabolomic data analysis and interpretation. *Nucleic acids research.* Jul 2009;37(Web Server issue):W652-W660. 10.1093/nar/gkp356 PMC2703878 19429898

[22] Dan Y, Zhang Y, Cheng L (2016). Hepatitis B virus X protein (HBx)-induced abnormalities of nucleic acid metabolism revealed by (1)H-NMR-based metabonomics. Scientific reports..

[23] El-Serag HB, Rudolph KL (2007). Hepatocellular carcinoma: epidemiology and molecular carcinogenesis. Gastroenterology..

[24] Ferlay J, Shin HR, Bray F, Forman D, Mathers C, Parkin DM. Estimates of worldwide burden of cancer in 2008: GLOBOCAN 2008. *International journal of cancer.* Dec 15 2010;127(12):2893-2917. 10.1002/ijc.25516 21351269

[25] Gao R, Cheng J, Fan C, et al. Serum metabolomics to identify the liver disease-specific biomarkers for the progression of hepatitis to hepatocellular carcinoma. *Scientific reports.* Dec 10 2015;5:18175. 10.1038/srep18175 PMC4674760 26658617

[26] Li H, Fan SF, Wang Y, Shen SG, Sun DX (2017). Rapid detection of small molecule metabolites in serum of hepatocellular carcinoma patients using ultrafast liquid chromatography-ion trap-time of flight tandem mass spectrometry. Analytical sciences : the international journal of the Japan Society for Analytical Chemistry..

[27] Ladep NG, Dona AC, Lewis MR, et al. Discovery and validation of urinary metabotypes for the diagnosis of hepatocellular carcinoma in West Africans. *Hepatology (Baltimore, Md.).* Oct 2014;60(4):1291-1301. 10.1002/hep.27264 24923488

[28] Lu Y, Li N, Gao L, et al. Acetylcarnitine is a candidate diagnostic and prognostic biomarker of hepatocellular carcinoma. *Cancer Res.* May 15 2016;76(10):2912-2920. 10.1158/0008-5472.CAN-15-3199 26976432

[29] Chen C, Chen DP, Gu YY (2015). Vascular invasion in hepatitis B virus-related hepatocellular carcinoma with underlying cirrhosis: possible associations with ascites and hepatitis B viral factors?. Tumour biology : the journal of the International Society for Oncodevelopmental Biology and Medicine..

[30] Li P, Huang W, Wang F, et al. Nomograms based on inflammatory biomarkers for predicting tumor grade and micro-vascular invasion in stage I/II hepatocellular carcinoma. *Bioscience reports.* Sep 25 2018. 10.1042/BSR20180464 PMC6239277 30254101

[31] Kurokawa T, Yamazaki S, Mitsuka Y, Moriguchi M, Sugitani M, Takayama T. Prediction of vascular invasion in hepatocellular carcinoma by next-generation des-r-carboxy prothrombin. *Br J Cancer.* Jan 12 2016;114(1):53-58. 10.1038/bjc.2015.423 PMC4716541 26679378

[32] Cox IJ, Aliev AE, Crossey MM, et al. Urinary nuclear magnetic resonance spectroscopy of a Bangladeshi cohort with hepatitis-B hepatocellular carcinoma: a biomarker corroboration study. *World journal of gastroenterology.* Apr 28 2016;22(16):4191-4200. 10.3748/wjg.v22.i16.4191 PMC4837436 27122669

[33] Shariff MI, Kim JU, Ladep NG (2016). Urinary metabotyping of hepatocellular carcinoma in a UK cohort using proton nuclear magnetic resonance spectroscopy. Journal of clinical and experimental hepatology..

[34] Shariff MI, Tognarelli JM, Lewis MR (2015). Plasma lipid profiling in a rat model of hepatocellular carcinoma: potential modulation through quinolone administration. Journal of clinical and experimental hepatology..

[35] Meiser J, Schuster A, Pietzke M, et al. Increased formate overflow is a hallmark of oxidative cancer. *Nat Commun.* Apr 10 2018;9(1):1368. 10.1038/s41467-018-03777-w PMC5893600 29636461

